# Baseline Muscle Mass Is a Poor Predictor of Functional Overload-Induced Gain in the Mouse Model

**DOI:** 10.3389/fphys.2016.00534

**Published:** 2016-11-15

**Authors:** Audrius Kilikevicius, Lutz Bunger, Arimantas Lionikas

**Affiliations:** ^1^Institute of Sport Science and Innovations, Lithuanian Sports UniversityKaunas, Lithuania; ^2^Animal & Veterinary Sciences, Scotland's Rural College, Roslin Institute BuildingMidlothian, UK; ^3^School of Medicine, Medical Sciences and Nutrition, College of Life Sciences and Medicine, University of AberdeenAberdeen, UK

**Keywords:** skeletal muscle, muscle fiber types, synergist ablation, hypertrophy, genetic background, inbred strains, congenic strains, consomic strains

## Abstract

Genetic background contributes substantially to individual variability in muscle mass. Muscle hypertrophy in response to resistance training can also vary extensively. However, it is less clear if muscle mass at baseline is predictive of the hypertrophic response. The aim of this study was to examine the effect of genetic background on variability in muscle mass at baseline and in the adaptive response of the mouse fast- and slow-twitch muscles to overload. Males of eight laboratory mouse strains: C57BL/6J (B6, *n* = 17), BALB/cByJ (*n* = 7), DBA/2J (D2, *n* = 12), B6.A-(*rs3676616-D10Utsw1*)/Kjn (B6.A, *n* = 9), C57BL/6J-Chr10^A/J^/NaJ (B6.A10, *n* = 8), BEH+/+ (*n* = 11), BEH (*n* = 12), and DUHi (*n* = 12), were studied. Compensatory growth of soleus and plantaris muscles was triggered by a 4-week overload induced by synergist unilateral ablation. Muscle weight in the control leg (baseline) varied from 5.2 ± 07 mg soleus and 11.4 ± 1.3 mg plantaris in D2 mice to 18.0 ± 1.7 mg soleus in DUHi and 43.7 ± 2.6 mg plantaris in BEH (*p* < 0.001 for both muscles). In addition, soleus in the B6.A10 strain was ~40% larger (*p* < 0.001) compared to the B6. Functional overload increased muscle weight, however, the extent of gain was strain-dependent for both soleus (*p* < 0.01) and plantaris (*p* < 0.02) even after accounting for the baseline differences. For the soleus muscle, the BEH strain emerged as the least responsive, with a 1.3-fold increase, compared to a 1.7-fold gain in the most responsive D2 strain, and there was no difference in the gain between the B6.A10 and B6 strains. The BEH strain appeared the least responsive in the gain of plantaris as well, 1.3-fold, compared to ~1.5-fold gain in the remaining strains. We conclude that variation in muscle mass at baseline is not a reliable predictor of that in the overload-induced gain. This suggests that a different set of genes influence variability in muscle mass acquired in the process of normal development, growth, and maintenance, and in the process of adaptive growth of the muscle challenged by overload.

## Introduction

Muscle mass can differ by more than two-fold in humans (Kim et al., 2002; Wolfe, 2006). One of plausible mechanisms behind that is that the numbers of muscle fibers among individuals of a similar age also differ over two-fold in both the arm (MacDougall et al., 1984) and the leg (Lexell et al., 1988) muscles. Such substantial variability in muscle mass can have an underappreciated impact on metabolism (Wolfe, 2006). It has been also reported that muscle mass in older adults has a positive association with longevity in older adults (Srikanthan and Karlamangla, 2014).

It is reasonable to hypothesize that such extensive differences in the quantity of muscle fibers may also affect the adaptation of muscle tissue to resistance training. Indeed, a response to a resistance programme can range between no hypertrophy and over 50% gain in muscle cross sectional area (Hubal et al., 2005). Such a variable outcome of a standardized and controlled training program is consistent with the findings that genetic predisposition plays a role in adaptive response (Thomis et al., 1998).

Laboratory mouse strains have been proven a useful model for studying the genetic effects on variety of traits including the skeletal muscle properties. Each strain represents a unique collection of alleles which is fixed and constitute a rather stable genetic background over generations. Comparison of relevant traits between animals of different strains reared under the same environmental conditions permits assessment of the role of the genetic background. Up to a 10-fold difference in muscle mass has been reported between inbred strains (Kilikevicius et al., 2013; Lionikas et al., 2013a,b). The number of fibers can differ by over two-fold (Lionikas et al., 2013b). Collectively that indicates a substantial impact of genetic variability on these traits.

In addition to the baseline properties, the ability to adapt to endurance type of training is also affected by genetic background; strains such as A/J show a very limited improvement in endurance capacity while the C57BL/6J and DBA/2J strains can progress substantially (Kilikevicius et al., 2013). Considering the extent of differences in muscle mass and fiber properties between inbred strains, it is reasonable to expect that their ability to adapt to resistance type of training would differ as well. However, to the best of our knowledge no studies have been carried out for testing such hypothesis.

The present study is aimed at examining the role of genetic background on muscle mass of the fast- and slow-twitch muscles and on their ability to adapt to an overload induced by synergist ablation. For the study we selected 8 strains: three high growth strains, BEH, BEH+/+, and DUHi (Bünger et al., 2001, 2004), three classical laboratory strains, C57BL/6J, DBA/2J, and BALBc/ByJ, and two strains, a congenic B6.A-(rs3676616-D10Utsw1)/Kjn and a consomic C57BL/6J-Chr10A/J/NaJ, derived from the C57BL/6J and A/J strains. The BEH strain carries a mutant allele of the myostatin gene, whereas a wildtype myostatin was introgressed in the BEH+/+ strain (Varga et al., 1997; Amthor et al., 2007). These two strains permit assessment of the role of a potent inhibitor of muscle development and growth on adaptation to the overload (McPherron et al., 1997). The DUHi strain exhibits an exceptional growth capacity and muscling which is unrelated to myostatin (Bünger et al., 2004). Mouse chromosome 10 harbors genes involved in myogenesis and metabolism, inclusion of the consomic and congenic strains permitted us to assess if strain variants might play a role in determining the baseline muscle bulk and also the adaptive growth. The slow-twitch soleus and fast-twitch plantaris muscles in the males of these strains were subjected to overload through a unilateral ablation of gastrocnemius.

## Materials and methods

### Animals

All animal procedures were approved by the Lithuanian State Food and Veterinary Service (Ref. # 0230). Mice were housed in the standard cages with *ad libitum* access to a standard chow diet (protein 29.8%, carbohydrate 56.7%, fat 13.4%) and tap water. The ambient temperature and humidity were maintained at 22–24°C and 40–60%, respectively with 12-h light/12-h dark cycle.

We selected a panel of strains capturing a broad range of genetic and phenotypic diversity in skeletal muscle traits. This permitted exploration of the role of genetic variability in compensatory growth and assessment of the influence of pre-existing morphological differences. There were three classical inbred strains, C57BL/6J (B6, *n* = 17), BALB/cByJ (BALB, *n* = 7), DBA/2J (D2, *n* = 12), which vary in muscle fiber properties and in adaptation to endurance training (Kilikevicius et al., 2013). A consomic C57BL/6J-Chr10^A/J^/NaJ (B6.A10, *n* = 8) and congenic B6.A-(*rs3676616-D10Utsw1*)/Kjn (B6.A, *n* = 9) strains derived from the B6 (host) and A/J strains (donor) were also included. The consomic strains, also known as chromosome substitution strains, are derived by a marker assisted introgression of an individual chromosome of a donor strain onto the background of a host strain (Singer et al., 2004; Ishii et al., 2011). A difference between a consomic and the host strain indicates that donor allele of a gene or genes residing on the chromosome affect the phenotype of interest. Further narrowing on the location of causative gene(s) can be achieved using congenic strains. We included a congenic B6.A strain which contains ≈1.5-Mb insert of A/J strain in the telomeric region of chromosome 10 (downstream of 127,454,593 bp) of the B6 strain genome (Johnson et al., 2012). Three high growth strains: BEH+/+ (*n* = 11), BEH (*n* = 12), and DUi (*n* = 12) were also selected. The BEH strain carries a 12 bp deletion in the myostatin gene (Varga et al., 1997), known as the *compact* allele, whereas a wildtype myostatin allele was introgressed in the BEH+/+ strain (Amthor et al., 2007). This pair of strains provides a model for assessing the role of myostatin in adaptation to the overload (McPherron et al., 1997). The DUHi exhibits an exceptional growth capacity and muscling not related to myostatin as it has been derived from a different founder population than the BEH strain (Bünger et al., 2004).

Only males were used in the analyses to eliminate the sex-related differences in muscle fiber properties such as cross-sectional area and proportion of the oxidative fibers (Carroll et al., 2012). These sex differences would affect muscle weight and might also contribute to variability in the adaptive response to overload reducing the statistical power for capturing strain-dependent effects.

### Synergist ablation procedure

To explore the compensatory growth of soleus and plantaris muscles, a unilateral ablation of gastrocnemius was performed as described earlier (Minderis et al., 2016). Briefly, adult mice (99 ± 3 day old) were anesthetized with intraperitoneal injection of ketamine/xylazine [100 mg (Richter Pharma AG, Austria)/10 mg (Eurovet Animal Health B.V., Netherlands) per kg] cocktail. Under anesthesia, a randomly selected hind limb was shaved and wiped with 70% ethanol. A longitudinal incision through the skin and fascia was made on the lateral side to expose the calf muscles. When the surrounding fascia and muscles were gently separated from the gastrocnemius, two-thirds of the distal part of the gastrocnemius was removed. The incision of fascia and skin were stitched with 6-0 and 4-0 size surgical suture, respectively. After the procedure, animals were single housed. Post-surgical pain was relieved with subcutaneous injection of ketoprofen (50 μg/10 g, Richter Pharma AG, Austria) directly after surgery and 24 h later. The contralateral limb remained intact and served as the control.

Four weeks after surgery animals were weighed (Kern, 440-43N, Germany) and euthanized by cervical dislocation. The soleus and plantaris muscles were removed bilaterally and weighed to 0.1 mg accuracy on analytic balance (Kern, ABS 80-4, Germany). For relative comparison of muscle weight and compensatory enlargement, tibia length was measured with a sliding caliper (accuracy 0.1 mm, LiMiT, New Zeland) as an index of the size of the skeletal frame for muscle growth.

After measurements it emerged that some overloaded muscles appeared smaller than the control. This is not consistent with the stereotypic adaptive response to overload but might be an outcome of accidental blood vessel or nerve damage. Therefore such animals (B6, *n* = 3; B6.A, *n* = 1; B6.A10, *n* = 1) were not included in any analyses.

### Statistics

The effect of strain on muscle weight and on the compensatory growth (gain = weight in ablated limb/weight in contralateral control limb) was analyzed using a multivariate general linear model followed by the Tamhane's T2 *post hoc* test using SPSS statistical package (IBM SPSS Statistics 23). Size of the skeleton reflected by the length of the tibia, and body weight varied substantially among the strains (Table 1). To assess if the strain effects were muscle tissue-specific and not secondary to the size of the skeleton, the analyses were repeated with tibia length included as a covariate. To enable a *post hoc* analysis on tibia length-adjusted muscle weights and compensatory gains, residuals of these variables were obtained via linear regression on tibia length. Mean and SD are presented unless otherwise stated.

**Table 1:** **Body weight (BW) and bone length of male mice from 8 inbred strains**.

	**n**	**BW, g**	**Tibia, mm**
B6	17	27.0 ± 1.5	17.7 ± 0.3
B6.A	9	27.0 ± 1.0	17.6 ± 0.1
B6.A10	8	31.7 ± 3.5	18.1 ± 0.1
BALB	7	29.0 ± 1.6	17.3 ± 0.3
BEH	12	57.5 ± 2.9	19.3 ± 0.4
BEH+/+	11	48.0 ± 1.9	18.7 ± 0.2
D2	12	26.1 ± 2.1	16.7 ± 0.6
DUHi	12	76.0 ± 5.7	22.5 ± 0.4

Mean ± SD.

## Results

### Baseline muscle mass

**Figure 1:**
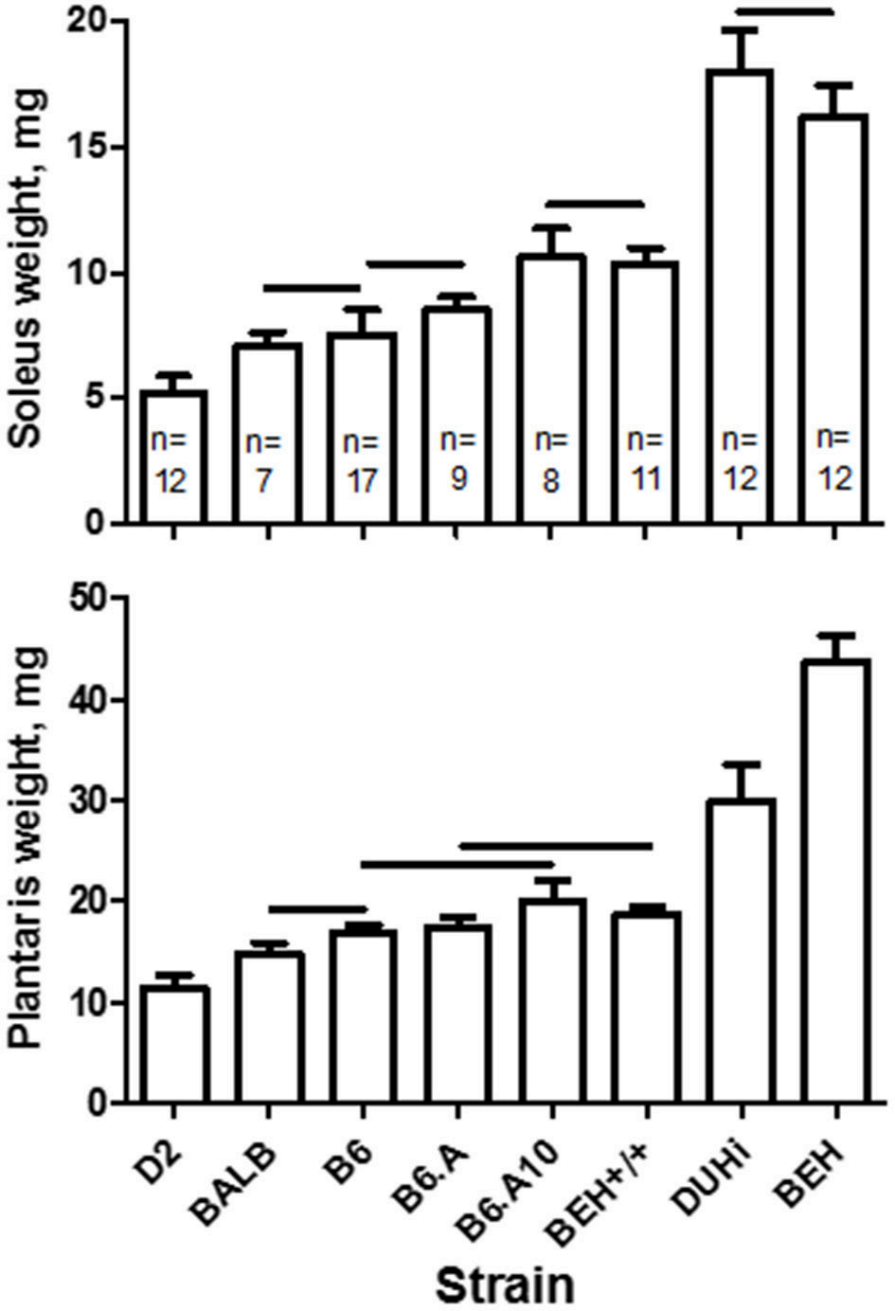
**Soleus (top) and plantaris (bottom) muscle weights in males of 8 inbred mouse strains**. Strain had a significant effect on soleus (*p* = 1.6 × 10^−49^) and plantaris (*p* = 8.3 × 10^−57^) muscles. *S*trains not connected by horizontal lines differ statistically significantly at *p* < 0.05. Sample size per strain is indicated (n) and applies to both muscles. Mean and *SD*.

The strain effect on muscle weight in the control limb was highly statistically significant (soleus, *p* = 1.6 × 10^−49^; plantaris, *p* = 8.3 × 10^−57^). The largest plantaris and soleus were observed in two high growth strains, BEH and DUHi, and the smallest in the D2 strain (Figure 1). Comparison of the three closely related strains, B6, B6.A, and B6.A10, revealed a higher soleus weight in B6.A10 mice (*p* < 0.05 in B6.A10 vs. two other strains). A similar trend was observed for the plantaris muscle compared to the B6 strain albeit it did not reach statistical significance (*p* = 0.05). The difference between two other closely related strains, BEH and BEH+/+, was profound, particularly in the plantaris muscle weight, exceeding two-fold.

The strain effect was retained following the adjustment for tibia length (soleus, *p* = 2.5 × 10^−19^; plantaris, *p* = 1.8 × 10^−44^). However, this adjustment revealed that the size of the skeleton substantially contributed to muscle weight differences. For instance, following adjustment, two other high growth strains, BEH+/+ and DUHi, did not differ in the adjusted soleus weight compared to the classical laboratory strains. However, the BEH strain remained significantly higher than any other strain (*post hoc* test *p* < 0.05 for both muscles), and the difference in soleus weight between the B6 and B6.A10 strains was retained (*p* < 0.05).

### Compensatory growth

**Figure 2:**
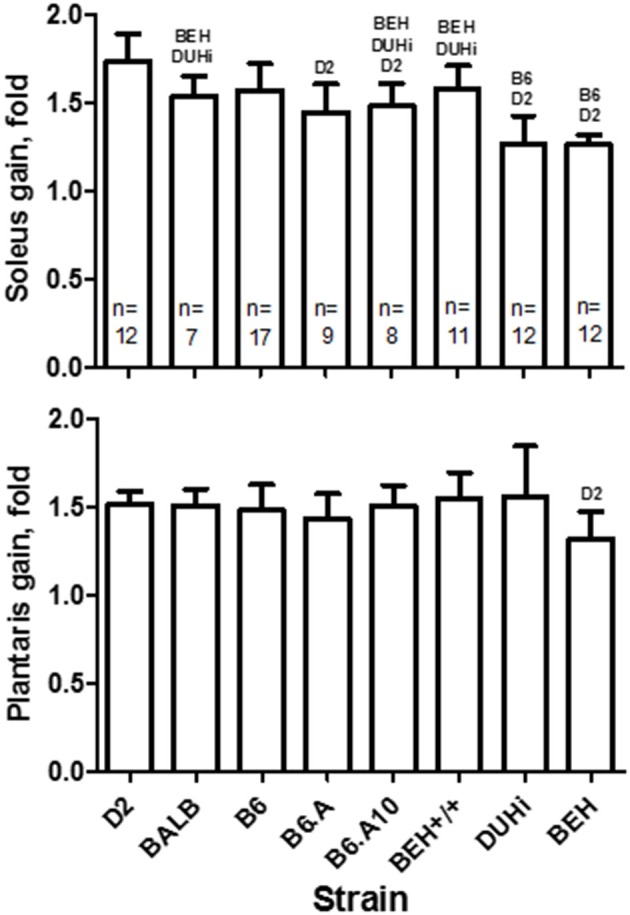
**Functional overload induced compensatory growth in soleus (top) and plantaris (bottom) muscles in males of 8 inbred mouse strains**. Strain exhibiting a statistically significant difference (*post-hoc* test *p* < 0.05) in the gain of the homologous muscle is indicated over the bar. Sample size per strain is indicated (n) and applies to both muscles. Mean and *SD*.

Four weeks of functional overload of soleus and plantaris muscles induced by ablation of the gastrocnemius resulted in ~1.5-fold weight gain in both muscles. However, strain had a significant effect on the degree of gain (*p* = 4.2 × 10^−13^ and *p* = 0.023 for soleus and plantaris, respectively). The D2 strain showed the largest increase in soleus, 1.7-fold, whereas the two high growth strains, BEH and DUHi, just 1.3-fold (Figure 2). Strain differences in the gain of the plantaris muscle were less prominent, yet the BEH again appeared the least responsive, 1.3-fold vs. 1.5-fold in the D2 strain (*post hoc* test *p* < 0.05). The BEH strain remained the lowest responder to the functional overload following the adjustment for tibia length (not shown); its adjusted gains in both soleus and plantaris muscles were lower compared to the closely related BEH+/+ strain (*post hoc* test *p* < 0.05 for both muscles). Variation in muscle weight at baseline could not explain the variation in the compensatory growth. The strain effect remained statistically significant for both soleus (*p* < 0.01) and plantaris (*p* < 0.02) muscles, and accounted for 12 and 5% of variation in the gain (based on adjusted *r*^2^), respectively, following inclusion of the baseline weight as a covariate. Comparison of the gain between soleus and plantaris muscles revealed the strain-specific differences (strain-by-muscle *p* = 3.4 × 10^−5^): the soleus gain exceeded that of plantaris in the D2 strain (*post hoc* test *p* < 0.02), but a reverse was observed in the DUHi strain (*post hoc* test *p* < 0.05).

## Discussion

We examined if accrual of muscle mass that occurs in the process of regular growth and tissue maintenance is predictive of the hypertrophic potential of the muscles subjected to overload. Phenotypically and genetically diverse panel of eight inbred mouse strains was used as a model. The main findings of our study were (*i*) that the overload-induced gain differs between the strains independently of muscle mass at baseline, (*ii*) that the extent of gain can differ between muscles in the strain-dependent manner, i.e., strain's superior gain in the slow-twitch soleus does not automatically predict a superior response of the fast-twitch plantaris or vice versa; and that (*iii*) life-long suppression of myostatin function is associated with reduced compensatory growth. An addition novel finding was that (*iv*) gene(s) on mouse chromosome 10 are involved in regulation of muscle mass (particularly slow-twitch muscle) with the A/J strain allele conferring an increase.

We first wanted to explore if the adaptive response to overload would differ between the strains, and if it did, whether that would occur in a manner predicted by such properties as the number of muscle fibers or irrespective of them. For instance, there is a considerable difference in the number of soleus fibers among the studied strains, ranging from ~760 in the D2 through ~1249 in the DUHi (Kilikevicius et al., 2013; Lionikas et al., 2013a,b). It is reasonable to hypothesize that, other factors remaining constant, more numerous fibers should confer greater gains due to a cumulative effect of fiber hypertrophy. However, such a model is not consistent with our findings (Figure 2). The D2 strain soleus increased 1.7-fold compared to ~1.3-fold for the DUHi or BEH (this strain's soleus contains ~1063 fibers; Kilikevicius et al., 2013; Lionikas et al., 2013a,b). Thus, it is not the number of fibers *per se*, that is positively associated with muscle mass gain. The strain effect, which explains 12 and 5% of variation in the gain of soleus and plantaris muscles respectively, implies that genetic variability between strains contributes to their differential response to the overload.

While individual variability in adaptation to resistance training in humans is well recognized (Hubal et al., 2005), it is much less known whether this is a global or muscle-specific phenomenon, and whether being a non-responder in the arm muscles predicts the same outcome in the leg muscles. Genetic variability combined with environmental factors is the underlying cause of individual differences. In a recent study, we found that an allelic variant of the *Met* gene conferring an increase in mouse soleus weight had a decreasing effect on the fast-twitch EDL and gastrocnemius muscles (Nicod et al., 2016). Hence, it is reasonable to hypothesize that the adaptive response might also differ between the muscles. The results of the present study support such notion demonstrating that the gain in soleus weight was a poor predictor of that in plantaris. For instance, while there was no difference in response between the soleus and plantaris in six strains (Figure 2), in the D2 strain, soleus appeared more responsive than plantaris, 1.7- vs. 1.5-fold gain, respectively, and vice versa in the DUHi strain, 1.3- vs. 1.6-fold. Thus, although all strains and both muscles conformed to a stereotypical adaptive response, the between-muscle differences suggest that the responder/non-responder phenomenon might be muscle-specific.

The discovery of myostatin as a potent inhibitor of muscle growth (McPherron et al., 1997) prompted development of pharmacological interventions targeting its pathways. However, trials conducted thus far have not delivered anticipated improvements in muscle mass (Wagner et al., 2008). Our recent study showed that functional overload results in attenuated hypertrophy of soleus muscle in BEH strain, carrying the *compact* allele of myostatin, compared to the BEH+/+, which has the *compact* allele replaced by a wildtype myostatin allele (Minderis et al., 2016). Thus, although the effects on muscle mass are profound when myostatin is ablated from the inception (McPherron et al., 1997), its role might differ under conditions where differentiated muscle is challenged to respond with adaptive growth. Furthermore, from our previous results it was not clear whether attenuation in hypertrophy is a general phenomenon or specific to the slow-twitch soleus muscle (Minderis et al., 2016). Findings of the present study showed that the compact allele similarly attenuates weight gain in the fast-twitch plantaris muscle (Figure 2).

Identification of causative genes is important for understanding the mechanisms underlying differences in muscle mass. The present study revealed a 41% larger soleus and 19% larger fast-twitch plantaris muscles in the B6.A10 compared to its host B6 strain. The B6.A10 consomic strain carries the A/J strain Chr 10 on otherwise the B6 strain background (Singer et al., 2004). The difference between the host (B6) and a consomic strain implies that the donor allele (A/J) of relevant gene(s) confers an increase in muscle weight compared to the allele of the host strain. A congenic B6.A strain carries a ≈1.5-Mb insert of the A/J strain in the telomeric region of chromosome 10 (Johnson et al., 2012). However, neither soleus nor plantaris weight of this strain differed from the B6 (Figure 1) indicating that the cause of the difference between the B6.A10 and B6 strains resides outside this congenic region. Such interpretation is also consistent with the soleus weight difference between the B6.A10 and B6.A strains (Figure 1). Additional studies will be required to determine the identity of causative gene(s). However, it emerges that allelic variants of that latent gene(s) conferred a similar compensatory growth between the B6.A10 and B6 strains.

In summary, quantitative differences in the gain of muscle mass induced by overload are genetic background- and muscle-dependent. Muscle mass at baseline is also influenced by genetic background and might result in extensive phenotypic differences. However, genetic background conferring an advantage in muscle mass at baseline does not ensure a similar advantage over other backgrounds in adaptive growth. This applies when comparing vastly different backgrounds (e.g., D2 and DUHi strains), and genetic backgrounds where differences are restricted to <3% of the genome (e.g., B6 and B6.A10 strains) or just to one gene (BEH and BEH+/+). We conclude that different sets of genes influence variability in muscle mass acquired in the process of normal development, growth and maintenance, and in the process of adaptive growth of the muscle challenged by overload.

## Author contributions

AL designed the study, LB provided BEH, BEH+/+, and DUHi mice, AK carried out experiments and collected data, AL and AK analyzed the data and wrote the manuscript, AK, LB, and AL edited the manuscript.

## Funding

This research was funded by the European Social Fund under the Global Grant measure. Grant VP1-3.1-ŠMM-07-K-02-057 was awarded to AL.

### Conflict of interest statement

The authors declare that the research was conducted in the absence of any commercial or financial relationships that could be construed as a potential conflict of interest.
